# A Novel Predictive Model Utilizing Retinal Microstructural Features for Estimating Survival Outcome in Patients with Glioblastoma

**DOI:** 10.21203/rs.3.rs-4420925/v1

**Published:** 2024-05-17

**Authors:** Rebekah Smith, Ranjit Sapkota, Bhavna Antony, Jinger Sun, Orwa Aboud, Orin Bloch, Megan Daly, Ruben Fragoso, Glenn Yiu, Yin Allison Liu

**Affiliations:** 1. School of Medicine, University of California, Davis; 2. Institute of Innovation, Science & Sustainability, Federation University Australia, Mt Helen, Australia; 2. Institute of Innovation, Science & Sustainability, Federation University Australia, Mt Helen, Australia; 4. Department of Neurology, University of California, Davis; Department of Ophthalmology & Vision Science, University of California, Davis; Department of Ophthalmology & Vision Science, University of California, Davis

## Abstract

Glioblastoma is a highly aggressive brain tumor with poor prognosis despite surgery and chemoradiation. The visual sequelae of glioblastoma have not been well characterized. This study assessed visual outcomes in glioblastoma patients through neuro-ophthalmic exams, imaging of the retinal microstructures/microvasculature, and perimetry.

A total of 19 patients (9 male, 10 female, average age at diagnosis 69 years) were enrolled. Best-corrected visual acuity ranged from 20/20–20/50. Occipital tumors showed worse visual fields than frontal tumors (mean deviation − 14.9 and − 0.23, respectively, p < 0.0001). Those with overall survival (OS) < 15 months demonstrated thinner retinal nerve fiber layer and ganglion cell complex (p < 0.0001) and enlarged foveal avascular zone starting from 4 months post-diagnosis (p = 0.006). There was no significant difference between eyes ipsilateral and contralateral to radiation fields (average doses were 1370 cGy and 1180 cGy, respectively, p = 0.42). A machine learning algorithm using retinal microstructure and visual fields predicted patients with long (≥ 15 months) progression free and overall survival with 78% accuracy.

Glioblastoma patients frequently present with visual field defects despite normal visual acuity. Patients with poor survival duration demonstrated significant retinal thinning and decreased microvascular density. A machine learning algorithm predicted survival; further validation is warranted.

## Introduction

Glioblastoma is a primary central nervous system (CNS) tumor classified by the World Health Organization (WHO) as a grade IV astrocytoma. It constitutes 14.3% of all primary CNS tumors and is the most prevalent (49.1%) malignant brain tumor [1]. A comprehensive global meta-analysis of primary CNS tumors indicates a worldwide prevalence of glioblastoma at 17.7% [2]. Despite its lower incidence compared to common cancers such as lung, breast, prostate, and colon [3], glioblastoma has an exceptionally grim prognosis, with an overall survival (OS) of approximately 15 months [4]. Biomarkers are essential for monitoring the treatment response and predicting outcomes, guiding patient care and counseling [5].

The ophthalmic features of glioblastoma patients have not been well studied. Prior investigations have revealed that patients with glioblastoma experienced lower health-related quality-of-life, both physically and mentally [6]. Visual disturbance as a presenting symptom of glioblastoma occurred in less than 5% of cases within 6 months before diagnosis [7]. A recent literature review reported that most neuro-ophthalmic sequelae in CNS neoplasms are associated with tumor location [8]. In cases of glioblastoma occurring as a malignant optic glioma, visual loss with or without pain was the predominant presenting concern [9–11]. However, for patients with cerebral glioblastoma, the impact on vision is less understood.

Optical coherence tomography (OCT) and OCT angiography (OCTA) are bioimaging techniques with the potential to assist in the monitoring of visual sequelae of brain tumors. OCT/OCTA captures two-dimensional images of retinal microstructure. The technology relies on low-coherence interferometry with infrared wavelengths (700–1,300 nm) and generates spatial resolution of 1–2 um [12]. There is a growing understanding that OCT/OCTA reveals how the retinal cell layers respond to CNS structural changes from injury through retrograde trans-synaptic degeneration (RTSD) [13–15]. Existing research correlated ganglion cell layer (GCL) thinning with RTSD following ischemic stroke [16]. Similarly, CNS changes in multiple sclerosis manifest in the retina as reduced optic nerve head perfusion seen with OCTA [17]. Additionally, research in Alzheimer’s type dementia (AD) reveals significantly lower vascular density in retinal zones and an enlarged foveal avascular zone (FAZ) using OCT/OCTA [18]. Ultimately it has been demonstrated that analysis of the retina with OCT/OCTA is an effective means of studying the CNS.

As an emerging tool in brain tumor research, machine learning (ML) has successfully predicted therapy response to laser interstitial thermal therapy with a first order dynamics algorithm [19]. It has also been used to predict tumor segmentation and the location of tumor recurrence following standard treatment using multi-modal fusion and nonlinear correlation learning [20]. Another investigation focused on how DNA repair functions can be used to generate an individualized model of 1, 2, 3, 5, and 10 years survival and recurrence rates with the LASSO-COX algorithm for patients with low-grade glioma [21].

We hypothesize that serial neuro-ophthalmic exams and advanced ophthalmic imaging can assess visual outcome and assist in the prediction of survival outcomes. This study aims to investigate how glioblastoma, and its treatment, affect vision by observing neuro-ophthalmic exams and measuring retinal structural and vascular changes using OCT/OCTA. We additionally seek to determine whether survival outcome can be predicted by analyzing this ophthalmic data using machine learning models.

## Methods

### Study participants

Patients with pathology-confirmed glioblastoma were recruited from the University of California, Davis Comprehensive Cancer Center (UCDCCC). Normal controls were recruited through clinic at the University of California, Davis Eye Center. The controls were not age or sex matched. The study design was approved by the UC Davis Institutional Review Board and adhered to the tenets of the Declaration of Helsinki (IRB #1923252–1).

Data were collected from March 3, 2020, through October 12, 2023. Eligibility criteria included: biopsy confirmed diagnosis of glioblastoma, ≥ 18 years old, and at least one visit to the neuro-ophthalmology clinic. Exclusion criteria consisted of a known history of glaucoma or age-related macular degeneration, dense media opacity precluding measurements, history of ocular trauma or concomitant ocular diseases, uncontrolled diabetes, or hypertension. For control participants, inclusion criteria included patients with a normal eye exam. Exclusion criteria for control patients included a history of glioblastoma or intracranial mass.

All patients were treated according to standard of care (SOC) protocol [22]. Eligible patients were consented and enrolled by certified clinical research coordinators. Oncology data included clinical demographics and treatment information such as age at diagnosis, date of diagnosis, resection dates, tumor location and markers, SOC protocol intervention timelines, date of second-look surgery, and date of death if applicable.

### Neuro-ophthalmic exam and imaging

All participants received serial neuro-ophthalmic examinations, including standard assessment Snellen best corrected visual acuity (BCVA), intraocular pressure (IOP), color perception using Ishihara color plates, pupil exam, ocular motility and alignments, examination of the anterior segment and fundus. OCT of the optic disc and macula were performed using Zeiss Cirrus (Carl Zeiss, Inc) for retinal nerve fiber layer thickness (RNFL) and ganglion cell/inner plexiform layer thickness (GCIPL). OCTA images were captured using the Avanti Optovue OCTA system (Optovue, Inc). Measurements were automated using the manufacturer’s software (Optovue RTVue) from 2 OCT images per eye. Data outcomes collected included microvascular densities of the radial peripapillary capillaries (CapRPC), internal limiting membrane-inner plexiform layer (ILM-IPL), inner plexiform layer-outer plexiform layer (IPL-OPL), and foveal avascular zone (FAZ). The Humphrey visual field Analyzer 3 (Zeiss, Inc.) was used to collect visual fields mean deviation (HVF MD).

### Retinal dosing calculations

In the RayStation software, retinal contours were delineated to facilitate retrospective dose calculations. Each retina was outlined as the posterior half of the eye wall, with a thickness of 3 mm. Treatment plans for all cases employed a volumetric arc technique (VMAT) for administering the treatment. The dose parameters considered included the mean dose to each retina and the maximum dose to 0.03% of the retinal volume.

### Data analysis

The data were then processed and analyzed with Microsoft Excel, and Prism GraphPad (Version 10.1.0). Measurements entered as Snellen BCVA were automatically converted to Logarithm of the Minimum Angle of Resolution (LogMAR) values using the Excel equation described by Tiew, Lim, and Sivagnanasithiyar [23]. The time since diagnosis for the neuro-ophthalmology visit was grouped into 0–3 months, 4–6 months, 7–9 months, 10–12 months, and 13–23 months intervals. Data points for each eye were treated as repeated measurements for the study participant. The PivotTable function in Excel was used to calculate average values for visual data and OCT and OCTA measures within specific groups. The data were then transferred to Prism GraphPad to create visual summaries of the data. Average values were calculated for BCVA, HVF MD, and IOPs at each time range since diagnosis. Similarly, data was calculated for patients with OS < 15 months or OS ≥ 15 months at each time range post-diagnosis, which is the standard duration of survival in patients with glioblastoma. Finally, average data for eyes contralateral and ipsilateral to tumors were also ≥ calculated for each time range. Kaplan-Meier analysis was used to calculate progression free survival (PFS) and OS. Two-sided p values were generated using Microsoft Excel and Prism (Version 10.1.0). The P values were not adjusted because multiple comparisons were not made.

### Machine learning algorithm

Initial data exploration was conducted using correlation analysis to identify relationships between the various clinical features and the overall survival and event free survival of the patients. This step was crucial for understanding the data structure and guiding subsequent analysis. Next, partial least squares discriminant analysis (PLSDA) was employed to predict two survival metrics: progression free survival (PFS, ≥ 15 months) and overall survival (OS, ≥ 15 months). This method distills the data into components that best explain the variance related to the outcomes [24]. The choice of PLSDA for our predictive modelling is rooted in its inherent suitability for classification problems, especially when handling features that may exhibit multicollinearity, e.g., the RNFL and GCC. The models were trained and tested using a leave-one-patient-out cross-validation approach. The performance of the models was evaluated using overall accuracy and were also visualized using a confusion matrix. The contribution of each individual feature in the PLSDA models was also computed and visualized to assess the stability of the models.

## Results

### Patient demographics and tumor characteristics

A total of 23 patients with glioblastoma who received neuro-oncology care at the UCDCCC were identified during the study period. Four patients were excluded due to lack of neuro-ophthalmic examinations. A total of 38 eyes from 19 patients were included. Median time from tumor diagnosis to first eye examination was 3.6 months (ranging 0–15 months).

**Figure 1:**
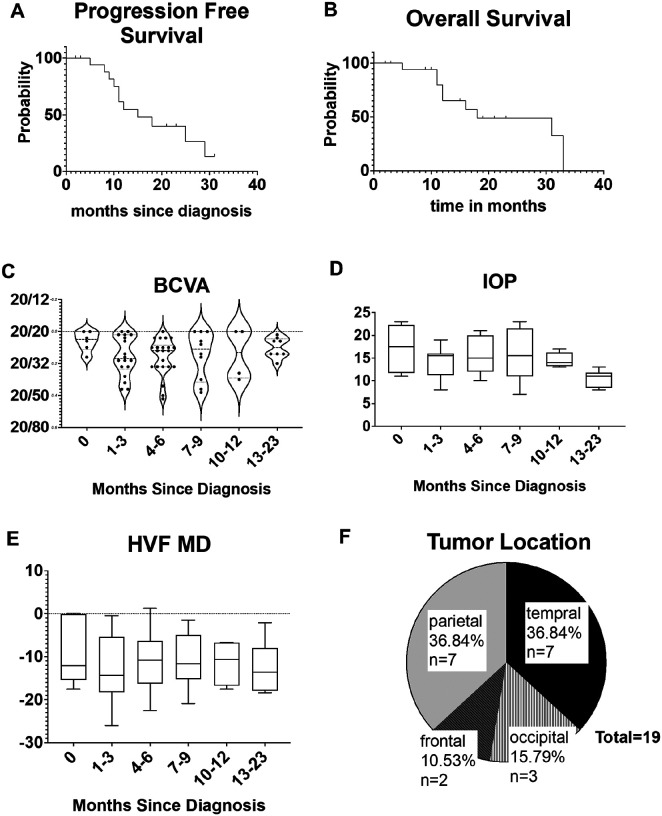
Visual characteristics and survival in a cohort of patients with glioblastoma. A) Progression free survival (PFS). B) Overall survival (OS). C) Best corrected visual acuity over time. D) Intraocular pressure (IOP) measurements over time. E) Humphrey visual field mean deviation (HVF MD) over time. F) Distribution of tumor location.

Table 1 contains patient demographics, tumor characteristics, and major events in their treatment courses. A total of 8 patients had radiographic progression (one with biopsy confirmed pseudoprogression (PsP) and 7 with biopsy confirmed recurrence. Nine patients died during the study period. The median PFS was 15 months (ranging 2–31 months) (Fig. 1A), and the median OS was 18 months (ranging 2–33 months) (Fig. 1B).

### Neuro-ophthalmic exam and association with tumor location

The BCVA of this cohort was better than 20/40 throughout the study period (Fig. 1C). Four patients had BCVA worse than 20/40, an equivalent of greater than 0.3 in LogMAR. The other fifteen patients had BCVA equal to or better than 20/40 throughout the study period. IOP of all subjects ranged from 7–23mmHg across the study period (Fig. 1D).

Most patients had moderate to severe visual field defects with HVF MD worse than − 2.0 dB (Fig. 1E). Only five patients had at least one normal visual field exam with HVF MD of −2.0 dB or better. Visual field defects were associated with tumor locations, with the most significant impairments observed in occipital tumors, demonstrating an average HVF MD of −14.9 dB, followed by temporal tumors at −11.5 dB, and parietal tumors at −10.2 dB. Frontal tumors exhibited the best visual field outcome with an average normal HVF MD of −0.23 dB (p < 0.0001). Figure 1F shows the distribution of each major tumor location.

### Retinal thinning and decreased microvascular density in patients with poor survival

**Figure 2:**
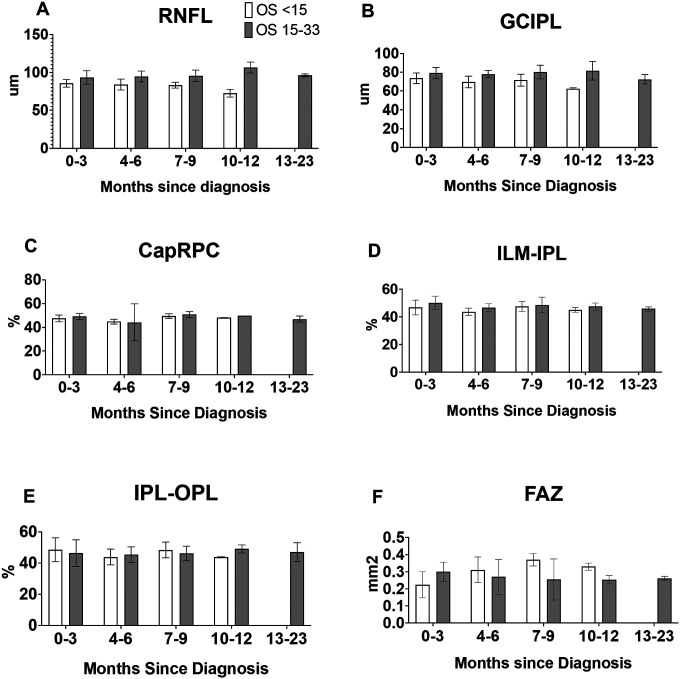
Comparison of retinal features in patients of glioblastoma demonstrating trends of retinal thinning and enlarged foveal avascular zone in patients with shorter progression free survival. A) retinal nerve fiber layer (RNFL) thickness, B) ganglion cell inner plexiform layer (GCIPL) thickness, C) radial peripapillary capillaries (CapRPC) measurement, D) inner retina, E) outer retina, and F) foveal avascular zone (FAZ) measurement over time.

The average RNFL thickness in patients with OS < 15 months was lower than that of patients in the OS 15–33 months group (p < 0.0001) Fig. 2A. This difference was observed at 0–3 months (p = 0.0132), 4–6 months (p = 0.0031), 7–9 months (p = 0.0047), and 10–12 months (p = 0.0040). The average GCC thickness in the OS < 15 months group was lower than that of the OS 15–33 group (p < 0.0001) (Fig. 2B). This difference was observed at 0–3 months (p = 0.0287), and 4–6 months (0.0012), but not at 7–9 months (p = 0.0526), or 10–12 months (p = 0.0620). Patients with long and short OS had the same CapRPC measurements on average (p = 0.8220) (Fig. 2C). For ILM-IPL, patients with long and short OS averaged the same measurements (p = 0.0836) (Fig. 2D). Similarly for IPL-OPL, patients with long and short OS averaged the same measurements (p = 0.7980) (Fig. 2E). When comparing all times post diagnosis for FAZ the OS < 15 group averaged the same as the OS 15–33 group (p = 0.4337) (Fig. 2F). However, at all times greater than four months post diagnosis, the average FAZ of patients with short OS was greater than patients with long OS (p = 0.0062). Despite this, there was not a consistent trend in FAZ. At 0–3 months FAZ was smaller in patients with short OS (p = 0.0260), at 4–6 months there was no difference between long and short OS (p = 0.3280). At 7–9 months the FAZ was smaller in patients with long OS (p = 0.0490), and at 10–12 months, there was no difference between long and short survival (p = 0.0782).

#### Radiation dosing has no measurable effect on the retina or visual acuity.

**Figure 3:**
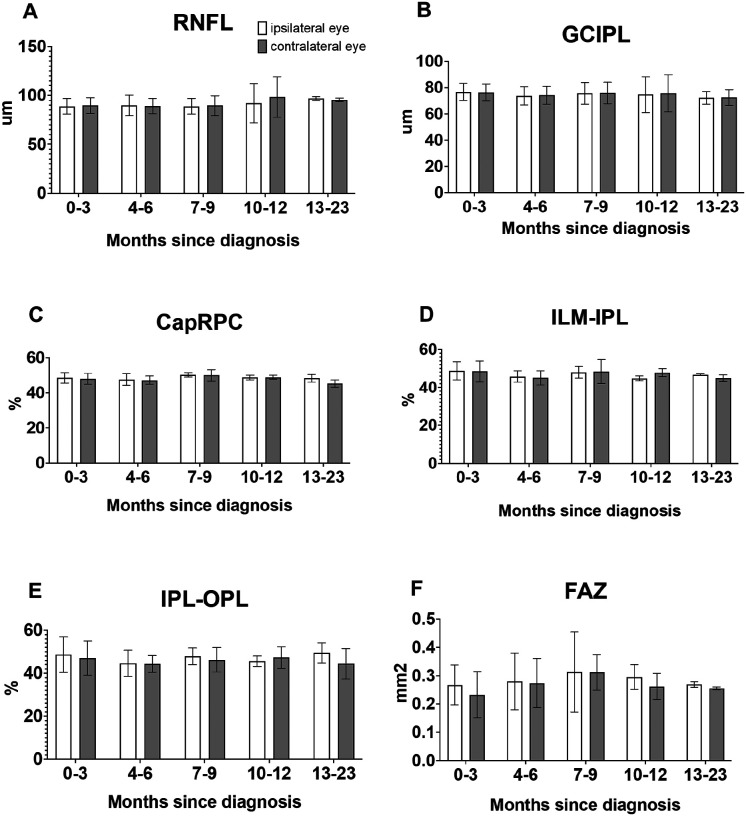
Comparison of retinal features between eyes ipsilateral and contralateral to radiation fields. A) retinal nerve fiber layer (RNFL) thickness, B) ganglion cell inner plexiform layer (GCIPL) thickness, C) radial peripapillary capillaries (CapRPC) measurement, D) inner retina, E) outer retina, and F) foveal avascular zone (FAZ) measurement over time.

No significant differences were observed in average retinal architecture and microvasculature between eyes that were ipsilateral and contralateral to radiation fields throughout the study period, nor was there appreciable differences in visual acuity. Eyes that were ipsilateral to radiation fields had average RNFL measurements of 89, 90, 89, 92, and 97μm while eyes contralateral to radiation fields had average RNFL of 90, 89, 90, 98, and 96μm at major post-diagnosis times (p = 0.9079) (Fig. 3A). For GCIPL, ipsilateral eyes had average measurements of 77, 74, 76, 75, 72μm while contralateral eyes had average GCIPL of 76, 74, 76, 76, and 73μm at major post-diagnosis times (p = 0.9201) (Fig. 3B). For CapRPC eyes ipsilateral to radiation fields had average measurements of 49, 48, 50, 49, 48% while contralateral eyes had average CapRPC of 48, 47, 50, 49, and 45% at major post-diagnosis times (p = 0.4691) (Fig. 3C). For the ILM-IPL ipsilateral eyes averaged 49, 46, 48, 45, and 47% while contralateral eyes averaged 49, 45, 48, 48, and 45% at major post-diagnosis times (p = 0.9550) (Fig. 3D). For IPL-OPL ipsilateral eyes averaged 49, 45, 48, 46, 50% and contralateral eyes averaged 47, 44, 46, 47, 44% at major times post-diagnosis (p = 0.7213) (Fig. 3E). For FAZ ipsilateral eyes averaged 0.267, 0.280, 0.313, 0.295, 0.269mm^2^ while contralateral eyes averaged 0.232, 0.273, 0.312, 0.262, and 0.255mm^2^ at major times post-diagnosis (p = 0.2585) (Fig. 3F). In terms of the BCVA LogMAR, ipsilateral eyes averaged 0.10, 0.14, 0.16, 0.15, and 0.11 while contralateral eyes averaged 0.13, 0.16, 0.13, 0.13, and 0.09 at major times post-diagnosis (p = 0.8330). The average mean radiation dosing for eyes ipsilateral to radiation fields was 1368 cGy (314–2987 cGy) and for contralateral eyes was 1180 cGy (406–1925 cGy) (p = 0.4166). The average max radiation dosing for ipsilateral eyes was 2014 cGy (419–4187 cGy) and for contralateral eyes was 579 cGy (640–2578 cGy) (p = 0.2843).

### ML model predicts PFS, OS

**Figure 4:**
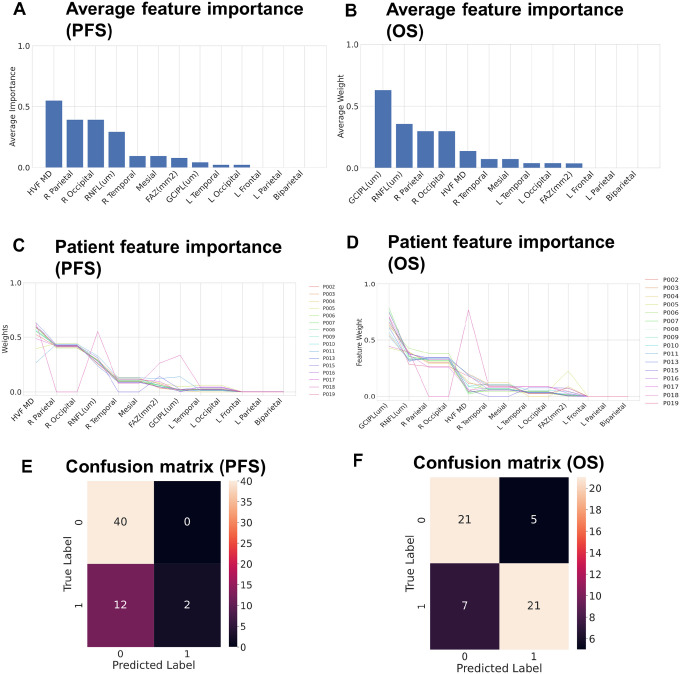
Machine learning model using neuro-ophthalmic features to predict survival outcome. A) Average feature importance in PLS-DA Model (PFS), B) average feature importance in PLS-DA Model (OS), C) individual patient feature importance in PLSDA Model (PFS), D) individual patient feature importance in PLSDA Model (OS), E) confusion matrix for prediction accuracy in computed models (PFS), F) confusion matrix for prediction accuracy (OS).

The PLSDA model was built using RNFL, HVF MD, and FAZ measurements taken from imaging captured throughout patients’ follow-up course, and location of the tumor (lobe and hemisphere) achieved an overall accuracy of 78% for PFS and OS from the initial diagnosis. For PFS, the location of the tumor along with RNFL thickness was found to have the highest weights, i.e., these features captured the covariance between the dependent variables and the independent variable. The feature weights were also analyzed for each of the models built using the leave-one-patient-out approach (see Fig. 4C and D), which were observed to be stable with few outliers. In the model that predicted OS, the GCIPL, RNFL and location of the tumors were found to have the highest feature weights. The model’s best performance was dependent on the inclusion of all features, including those with lower feature weights. The confusion matrix showed a stable classification with equal true positives and false positives (see Fig. 4, E and F). A standard regression demonstrated no linear relationship between OS and RNFL or OS and GCIPL.

## Discussion

In this study, we analyzed data through neuro-ophthalmic examination and OCT/OCTA of the retina for predictive modeling of survival in patients with glioblastoma. Our data suggest that patients with glioblastoma sustain significant peripheral vison loss while maintaining good central vision. Additionally, optic nerve thinning and microvascular density reduction, serving as markers for RTSD [13–15], are strong predictors of survival duration.

Vision is critically important for patients with terminal disease. The ability to see art, nature, caregivers, and adequate ambient light has been shown to greatly improve end of life care as noted by a comprehensive review [25]. In contrast with previous reports of glioblastoma causing decreased visual acuity in patients with optic nerve gliomas [10], our study demonstrated that almost all non-optic pathway glioblastoma patients fortunately had preserved central vision. In addition, our study result is similar to previous studies demonstrating an association between brain tumors in the temporal, parietal and occipital lobes with significant visual field defects [26].

Previous research suggests that brain parenchymal alternations cause RTSD to the retina and optic nerve. Specific examples include ischemic stroke [16], AD [18], multiple sclerosis [17], and Dutch-type hereditary cerebral amyloid angiopathy (D-CAA) [27]. OCT/OCTA plays a role in early detection of mild cognitive impairment and AD [18, 28, 29]. Carriers of D-CAA show thinner peripapillary RNFL once they become symptomatic, but not before [27]. Evidence for this comes from studies of retrochiasmal diseases causing retinal thinning and optic nerve damage as detected by OCT/OCTA. Thus, they serve as comparable models of visual pathway injury caused by brain tumors. Our study demonstrated similar result that suggest OCT/OCTA can be used to detect RTSD in glioblastoma patients over time.

Many biomarkers have been studied to guide management and continue to drive research in glioblastoma treatment. *MGMT* methylation in glioblastoma is a good prognostic marker because these tumors have a significantly better response to temozolomide therapy compared to unmethylated tumors [30]. Other studies have identified that MARCO-expressing macrophages, typically present in *IDH*-wild type glioblastomas only, are associated with worse prognosis [31]. Four additional genes, IFI30, HLA-DMA, P4HB and RCN1 are possible markers of prognosis and the focus of future studies [32]. Our study provided additional evidence that it is possible to use the neuro-ophthalmic exam and imaging biomarkers to predict glioblastoma prognosis. We found that a thinner RNFL was associated with reduced OS (p < 0.0001). Also, a thinner GCIPL was associated with reduced overall survival (p < 0.0001). These findings suggest that there is increased RTSD to the retina in glioblastoma with a faster rate of progression, conferring poorer prognosis. This possibility is supported by studies which have shown that thinning of the GCIPL and RNFL detected by OCT correlate with brain tumors causing mass effect symptoms on the optic chiasm and intracranial optic nerves [33].

Additionally, we observed that after four months post-diagnosis the average FAZ in the OS < 15 months group (0.333 mm^2^) is enlarged compared to the OS 15–33 months group (0.263 mm^2^) (p = 0.0062). Contrast this to a similar finding; eyes ipsilateral to a choroidal melanoma have an enlarged deep FAZ compared to the contralateral eye [34]. These findings suggest that malignant masses of the head can reduce vascularity in the retina.

Radiation Retinopathy (RR) is a recognized consequence of radiation dosing in the treatment of head and neck neoplasms. Following treatment, retinopathy can manifest either one month or up to 15 years later and is linked to a significant progressive decline in visual acuity [35]. The potential to compromise vision, and consequently a patient’s quality of life, is a crucial factor when assessing treatment options. A systematic review determined that RR predominantly occurs at doses exceeding 50 Gy [36]. Our patients received doses below this threshold, with an average maximum dose of 20 Gy for ipsilateral eyes and 6 Gy for contralateral eyes. We did not observe evidence of a significant decline in visual acuity, possibly indicating a correlation with dosing. Additionally, we acknowledge that through prolonged observation, signs of RR may become detectable; however, this proves challenging in patients with glioblastoma.

Predictive modeling is an actively developing field in brain tumor research. In this study, we introduce a predictive model based on PLSDA using data obtained in the neuro-ophthalmology clinic. Retinal features combined with tumor location provided the best performance showing a mean accuracy of 78% for PFS and OS prediction. Comparatively, the two best studies modeling survival as a continuous outcome report concordance index (c-indices) of 0.70 [37]. In 2017 a study that examined patient age, sex, race, MGMT methylation, performance status, resection extent, and tumor site used a Cox proportional hazard model to achieve a c-index of 0.695 ± 0.023 standard error for 1 year survival [38]. In 2018, another study employed similar inputs including patient age, sex, Karnofsky Performance Status, MGMT methylation, and subventricular zone tumor location classification through a Radiation Therapy Oncology Group nomogram to achieve a c-index of 0.70 at 12 months survival [39]. For time-to-event models, many studies report a c-index of 0.70 [37]. In 2020 a study employed 14 different machine learning models to learn from 13 factors within the following categories: surgical, demographic, socioeconomic, clinical, and neuro-radiographic to achieve a range of 66–70% concordance index across all models [40]. A c-index of 0.82 has been reported in a single study from 2013 which employed a multivariable analysis studying patient age, Eastern Cooperative Oncology Group (ECOG) performance status, and corticosteroids use [41]. While this represents the highest c-index in the literature we identified, the results have not been repeated since the original study in 2013. Our results indicate that ocular parameters combined with traditional patient demographics, tumor details and surgical features can generate high performing predictive models of survival in glioblastoma. Despite this, the false negative rate in our study for PFS was concerning (nearly 85%), indicating that the model erroneously predicted most samples as progression free. A larger study is needed to understand why the prediction of PFS is harder than OS, where we do not see a high false negative rate.

We also learned that of the features considered by the ML model, the characteristics associated with patient 18, the only patient with pseudoprogression, long survival and a particular type of glioblastoma (IDH wild type, MGMT methylated, EGFR positive, ATRX retained), had significant impact on the model’s predictions. These features include the HVF MD, FAZ, and occipital location of the tumor. These highlighted features give direction for future studies. In the continuation of this study, we will aim to refine our model towards distinguishing pseudoprogression from true tumor recurrence. This would significantly impact the field by reducing the burden of second-look surgery on patients following treatment, thus improving the quality of their lives. We believe that the identified biomarkers, including retinal nerve fiber layer thickness and foveal avascular zone, present a promising avenue for predicting survival outcomes. These findings hold great potential for clinicians in tailoring treatment strategies and counseling patients, ultimately improving the overall management and care of glioblastoma patients.

A major limitation of this study is the small sample size of participants, and eye data at specific times post-diagnosis. We are cognizant that the PLSDA model trained in this study is susceptible to overfitting and poor generalizability due to limitations in data validation and testing. This limitation reflects the natural course of the disease, as many glioblastoma patients opt for hospice care following or during initial treatment, leading them to understandably discontinue eye clinic visits for neuro-ophthalmic examinations. Furthermore, the fact that there was only one patient with pseudoprogression limits our ability to directly compare this disease outcome with true recurrence. In future studies we seek to expand our sample size by including additional patients with PsP to explore the predictive potential of neuro-ophthalmic markers.

## Conclusions

Visual field defects are the most common visual sequelae in patients with glioblastoma, while visual acuity remains relatively preserved. Optic nerve thinning and decreased retinal microvascular density are the most prominent outcome predictors for survival. A predictive model was established using features of visual field defects, retinal nerve fiber layer thickness, foveal avascular zone and tumor location demonstrating a 78% accuracy in predicting overall survival, whether better or worse than 15 months. A larger study is necessary to validate the results of this study.

## Figures and Tables

**Table 1:** Patient demographics and tumor information.

Patient	Age at Dx/gender	WHO grade	IDH	MGMT	EGFR	ATRX	XRT duration (w)	adjuvant TMZ status	Disease status at end of study
1	71/F	4	wild type	methylated	positive	retained	6.0	did not start due to thrombocytopenia	recurrence
2	47/F	4	wild type	methylated	positive	not done	6.3	completed C6D5	deceased
3	69/M	4	wild type	unmethylated	positive	retained	6.0	completed C6D5	recurrence, deceased
4	71/F	4	wild type	unmethylated	negative	retained	5.9	did not start due to thrombocytopenia	alive
5	68/M	4	wild type	methylated	not done	retained	6.1	completed C6D5	recurrence, deceased
6	52/F	4	wild type	unmethylated	positive	retained	2.9	completed C6D5	alive
7	59/F	4	wild type	methylated	positive	not done	6.0	Completed C3D5, stopped	recurrence
8	52/F	4	wild type	unmethylated	positive	retained	5.3	completed C3D5, ongoing	alive
9	60/M	4	wild type	unmethylated	not done	retained	6.4	completed C1D5, stopped	deceased
10	70/M	4	wild type	unmethylated	positive	retained	6.4	completed C4D5, stopped	recurrence, deceased
11	47/M	4	mutant	methylated	not done	lost	6.9	completed C6D5	alive
12	58/F	4	wild type	methylated	not done	not done	6.6	completed C6D5	recurrence, deceased
13	67/F	4	wild type	methylated	positive	retained	6.0	completed C3D5, stopped	deceased
14	76/M	4	mutant	methylated	positive	retained	6.0	completed C5D5, stopped	recurrence, deceased
15	74/M	4	wild type	unmethylated	positive	retained	5.6	completed C3D5, stopped	deceased
16	65/M	4	wild type	methylated	positive	retained	6.1	completed C4D5, ongoing	alive
17	73/F	4	wild type	methylated	positive	retained	5.3	completed C6D5	alive
18	55/M	4	wild type	methylated	positive	retained	6.0	completed C6D5	PsP, alive
19	71/F	4	wild type	unmethylated	positive	retained	6.4	opted not to begin	alive

## Data Availability

The data collected and used for analysis of this investigation can be found on the Harvard Dataverse Repository under the following citation; Smith, Rebekah; Liu, Yin Allison, 2024, “Retinal Microstructural Features for Estimating Survival Outcome in Glioblastoma Patients”, https://doi.org/10.7910/DVN/RVTV8Z, Harvard Dataverse, V1.
